# Life outcomes of being born with anorectal malformation: a systematic review of intersectional reporting, mental health co-morbidities, and psychosocial experiences in adulthood

**DOI:** 10.1007/s00383-026-06307-8

**Published:** 2026-02-21

**Authors:** Eloise Rane, Julia Bloom, Carys Chan, Paul Harris

**Affiliations:** 1 School of Allied Health, Sport, and Social Work, Griffith University, Brisbane, Australia; 2 Department of Management, Griffith Business School, Griffith University, Brisbane, Australia

**Keywords:** Anorectal malformation, Psychosocial functioning, Social work

## Abstract

**Purpose:**

Anorectal malformations exist across a spectrum of abnormalities involving the rectum, distal anus, genital and urinary tracts, and occur in approximately one in 5,000 live births. The purpose of this study was to investigate intersectional reporting, mental health co-morbidities, and psychosocial experiences of adults born with anorectal malformation.

**Methods:**

A systematic quantitative literature review was utilized to collect and analyze data. Articles were required to discuss population demographics, mental health comorbidities, or psychosocial experiences associated with anorectal malformation in adulthood. A total of 94 articles were found suitable for review.

**Results:**

Patient ethnicity, culture, sexuality, and spirituality were significantly underrepresented. Mental health co-morbidities such as anxiety and depression were discussed but rarely transitioned to intervention. Psychological challenges included psychosexual anxiety and limited professional knowledge. Sociological challenges included navigating health services and social settings. Literature prioritized continence outcomes that diminished psychosocial complexities.

**Conclusions:**

The intersectionality, mental health concerns, and psychosocial experiences of adults born with anorectal malformation remain largely unknown. Allied health professionals such as social workers can improve ongoing support provision, with interventions that enhance psychosocial functioning and emotional wellbeing.

## Introduction

### History of ARM

Descriptions of anorectal malformation (ARM) exist in ancient medical writings, whereby Aristotle (322–384 B.C.E) referenced anal atresia (currently known as imperforate anus) in an attempted but failed surgery to amend the condition via incision [1]. Treatments for ARM continued into the second century with Greek physician Soranos (98–138 C.E) describing a process of cutting through the perineal membrane, followed by dilation of the new opening [2]. From the early 1980’s to present day, treatment for ARM commonly involves the posterior sagittal anorectoplasty (PSARP). Developed by deVries and Peña (1982), the technique is founded upon full exposure of closely joined canals requiring separation [3]. When identified, surgical intervention for ARM most commonly takes place shortly after birth. However, the extant literature indicates a lack of transitional care occurs between pediatric and adult health services [4]. Therefore, despite life-saving medical advancements in recent decades; little consideration has been given to what outcomes those infants experience in present-day life as adults. Furthermore, how individuals are managing or coping with this condition, including the psychosocial stressors by which it is accompanied; remain distinctly unexplored [5–8].

### ARM classifications

The cause of ARM remains unknown; however, it is understood to be multifactorial in premise. Certain factors have been identified as paternal cigarette smoking, paternal exposure to exhaust gases or particulate matter, maternal fever during the first trimester of pregnancy, maternal (Body Mass Index [BMI] ≥ 25 kg/m^2^) prior to pregnancy, and one or more (first or second relation) family members with the condition [9]. In 1984 the ‘Wingspread Classification’ was created, aiming to standardise the assessment and recording of ARM sub-types, including corresponding surgical interventions [10]. The ‘Krickenbeck Classification’ followed in 2005, becoming a universal system for improved classification accuracy [11]. Recommendations from the new classification system included that patients be monitored for approximately ten years post-operatively. Therefore, long-term outcomes were predominantly forecast, rather than known, with the lived experience of the condition in adulthood remaining unexplored in the research [12]. The current study therefore examined the literature based on the premise of gaining a broad awareness of phenomenological complexities, exploring intersectional elements, mental health co-morbidities, and psychosocial experiences of the adult patient population.

## Methods and materials

### Research question

This systematic review aimed to discover, ‘What does the published literature report and prioritize regarding the intersectionality, mental health, and psychosocial experiences of adults born with anorectal malformation?’.

## Methodological framework

The analytical framework of the systematic review was underpinned by exploring elements of intersectionality in the extant literature [13]. Intersectionality recognizes the many different aspects of an individual’s identity overlap and intersect, and therefore, requires examination as a complex system. Previous research focused solely on achieving perceivably successful continence outcomes, examining this element in isolation of the person’s identity, mental wellbeing, and life experiences. However, a person’s continence does not exist in isolation from their life, nor can such an outcome define what success may mean for that individual. In this manner, the intersectional framework suggests that the collection and analysis of data be holistic in design. Categories intentionally addressed by the intersectional framework included: nationality, age, culture, socio-economic positioning, sexuality, relationship status, parenthood, language, spirituality, disability, education, employment, and mental health. This approach allowed the discovery of the extent to which intersectional elements were represented in the literature base, highlighting individuals who are potentially underrepresented, or underreported, in the published literature.

In addition to the intersectional framework and mental health co-morbidities, the inclusion of a psychosocial framework of challenges and supports enabled the effective collection, management, and analysis of datasets. The frameworks allowed ARM to be observed as both an intersectional element of one’s identity, and a psychosocial experience when navigating the world with a complex condition. For this reason, analysis remained attentive to the interconnectedness of the intersectional and psychosocial frameworks, recognizing that psychological factors influence well-being, which in turn affects social functioning. This approach broadened research beyond the previously singular and biologically focused understandings of the condition; that had minimized the psychological and sociological impacts of living with ARM in adulthood.

## Methods

**Fig. 1:**
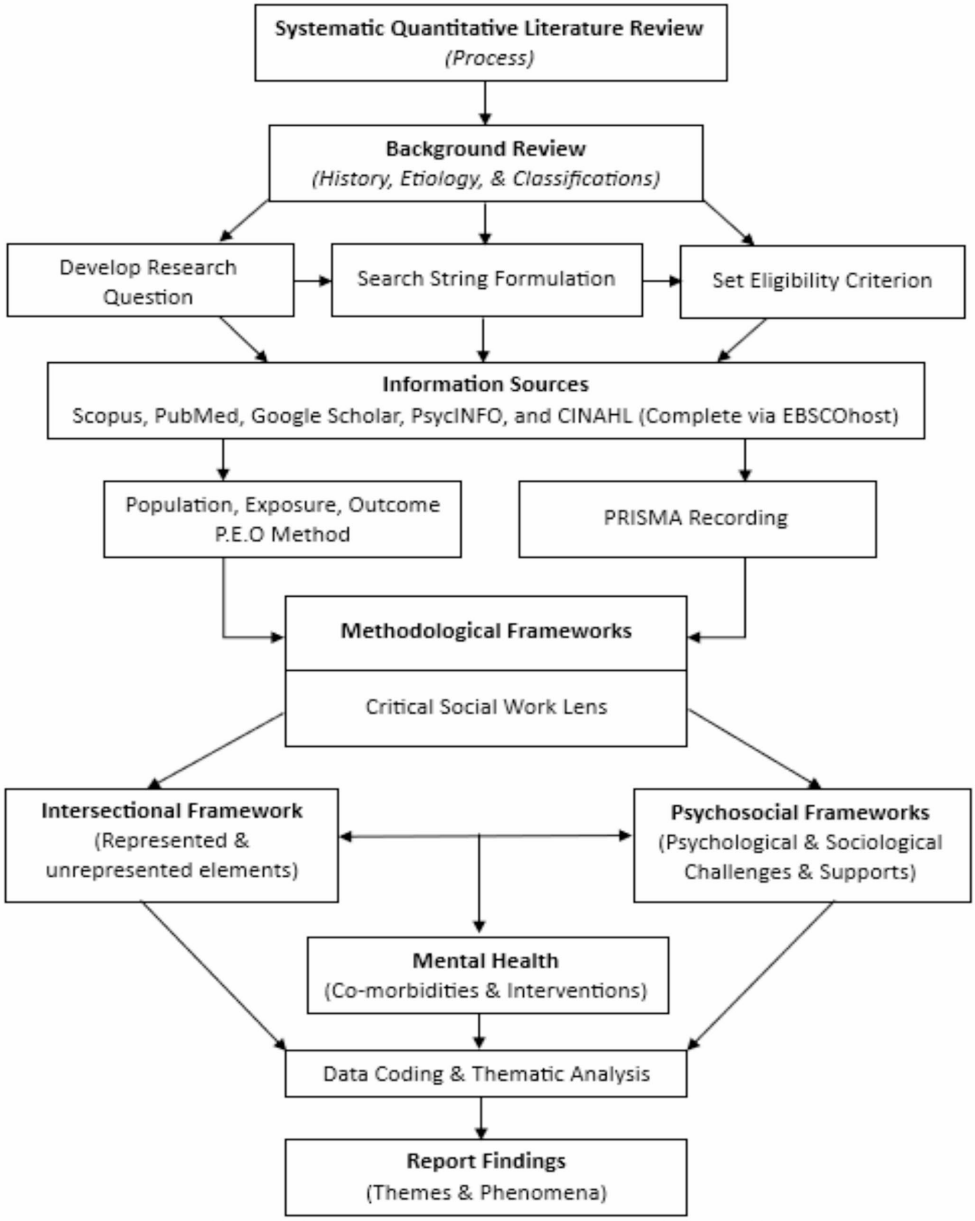
Flow chart of the systematic quantitative literature review process

A systematic quantitative literature review was employed to undertake this research [14]. Data from within the extant literature was systematically searched, extracted, and categorized within two separate datasets; constructed using an intersectional framework, and psychosocial framework. This method enabled data to be thematically coded and analysed across broad themes, and nuanced findings relevant to the research question. It also provided a reliable and reproducible quantitative assessment of the literature base and research gaps. A flow chart representing the complete systematic review process in provided in Fig. 1.

### Eligibility criteria

The set eligibility criteria for inclusion in the systematic review comprised articles which explored at least one of three broad areas aligned with the research question:

1. Articles which included a population that could be further analysed to assess intersectional representativeness; or.
2. Articles that discussed mental health co-morbidities of the condition, including any interventions utilised to support the adult ARM population; or.
3. Articles that reported on psychological or sociological challenges or supports expected or experienced in living with the condition in adulthood.

In addition, articles included for review were required to be original, peer-reviewed research, available in institutional databases, and full-text English. The search was not limited by publication date; to highlight themes and phenomena over time. Articles were thereupon discovered from the year 1986 to 2024.

### Information sources

Information sources for data collection included five electronic databases: Scopus, PubMed, Google Scholar, PsycINFO, and CINAHL (Complete via EBSCOhost). Searches of these five databases were performed across a one-week period from 23 June 2024 to 30 June 2024.

### Search string formulation

Search string formulation was intentionally broad, so as not to orientate results towards one domain (intersectional, psychological, sociological) over another. Using the Population Exposure Outcome (PEO) approach [15] enabled the domains to be inputted into the PEO mnemonic system as a population (adult patients), exposed to a phenomenon (born with ARM), and associated outcomes (psychological and sociological). Thereby, the following search string was applied: (“adult patient*”) AND (anorectal malformation*) and inputted into databases as: Database > No limitations applied > Search > All fields > “adult patient*” AND “anorectal malformation*”. No changes or filters were applied to settings, search fields, limitations, or language inputs between databases, ensuring reproducibility.

### Screening

**Fig. 2:**
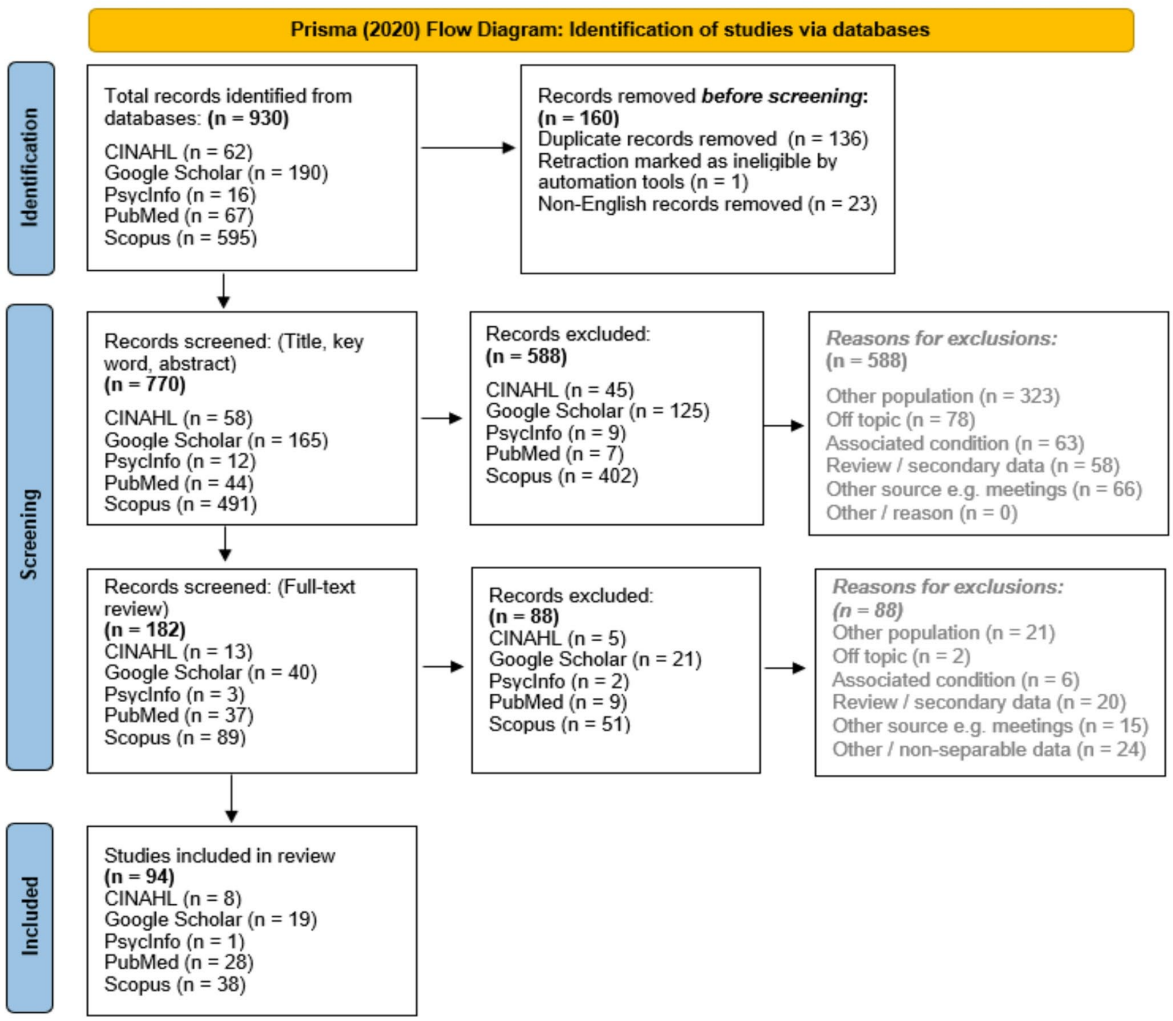
PRISMA (2020) flow chart of literature screening

The Preferred Reporting Items for Systematic Reviews and Meta-Analyses (PRISMA) was utilised to record screening cycles [16]. A total of 930 articles were located and screened, finding 94 eligible articles included for review. The PRISMA flowchart results are indicated in Fig. 2.

### Quality appraisal

**Fig. 3:**
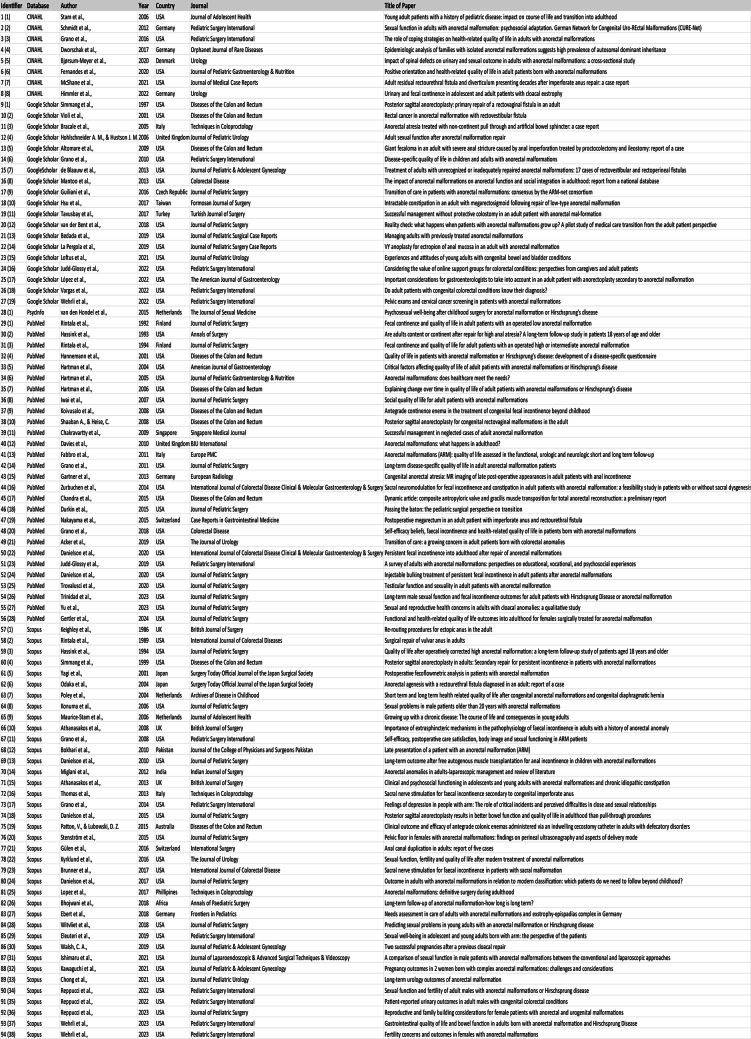
Summary table of 94 articles included in the systematic review

The Mixed Methods Appraisal Tool (2018) was adopted to complete article appraisal. However, quality appraisal alone did not warrant exclusion of articles, as overall score calculations are discouraged in context of differing approaches contributing subjectively to the overall field of interest [17]. The purpose of conducting the appraisal was instead exploratory in nature. All 94 articles were found feasible for review using the appraisal tool. Only one article adopted a mixed methods approach, five articles were qualitative in design, and the remaining (*n* = 88) were quantitative descriptive. There were no non-randomized or quantitative randomized controlled trials. The most prevalent methodological quality criterion not met was non-response biases in over half (*n* = 56) of the reviewed articles. This appeared due to non-reporting of response rates or sampling pools, requiring manual calculation to ascertain or an inability to do so. In addition to PRISMA recording, and the mixed methods quality appraisal; a summary table of the 94 articles included in the systematic review is also provided in Fig. 3.

### Dataset construction

Spreadsheets were developed using Microsoft Excel software, with collected data categorized in alignment with addressing the research question. The first spreadsheet, ‘Dataset 1.0’ inscribed patient demographics including: nationality, age, culture, socio-economic positioning, sexuality, relationship status, parenthood, language, spirituality, disability, education, employment, and mental health. The second spreadsheet, ‘Dataset 2.0’ inscribed psychosocial challenges and supports expected or experienced by adults living with ARM. Themes were identified using a block and file approach to data coding [18] allowing for information to be critically examined within the underpinning intersectional and psychosocial frameworks.

## Results

### Dataset 1.0: intersectional demographics

Intersectional elements covered nationality, age, culture, socio-economic positioning, sexuality, relationship status, parenthood, language, spirituality, disability, education and employment [13]. This critical lens elucidated visible and not visible populations, alongside exclusionary research practices embedded within the reviewed literature. More than half (*n* = 62) of the reviewed studies were conducted in North America; predominantly within the United States. Studies were largely cross-sectional in design, with a lack of longitudinal studies emerging as a consistent research limitation when representing the adult ARM cohort. This was despite long-term studies identified as being necessary in determining outcomes beyond pediatric presentation [19]. The limitations reported in (*n* = 33) 35% of studies included reportedly small sample sizes, selection bias, and missing data; oftentimes by way of employing incomplete hospital records.

### Nationality and age

The most reported nationality of patients was Italian in 15 studies, coinciding with ameliorated research and advocacy in the country [20]. This was followed by seven studies with Japanese patients, five with German patients, three each with Dutch and Indian patients, and two studies each with Pakistani, Australian, and Finnish patients. One study each existed with Swiss, Danish, Latino, Thai, French, Taiwanese, African, Guatemalan, Swedish, and Filipino patients. Conversely, seven studies homogenized patients as ‘predominantly Caucasian’, and the remaining 38 did not report on participant nationality or ethnicity. The age range of patients with ARM in the reviewed data spanned from 16 years of age to 71 years of age. However, the average age of patients within the review was 30.2 years; suggesting patients were returning to surgeons for clinical review long after the Krickenbeck (2005) index recommendation of 10 years post-operatively [11].

### Culture and sociodemographics

Culture and class systems were reported in only 18 articles. From these, eight discussed patients’ lack of social resources, health literacy, matters of family negligence, and resource limited environments as perceived impacts upon engaging with adult health services for their ARM. Occupational and insurance related concerns were mentioned in four of these articles, and four studies acknowledged the need for improved cultural and sociodemographic exploration in future research. Cultural obligations such as marriage and parental influence affecting the patient’s access and timing of access to health care were briefly mentioned in two articles. The remainder of reviewed articles (*n* = 76) did not report on culture or class systems that may be impacting patients within those studies and consequently, potential impacts upon navigating ARM in adulthood.

### Sex and sexuality

Female and male patients were combined for reporting purposes in 48.9% of the reviewed studies; despite perceivably increased differences between sexes in the adult cohort. Female-only studies totalled 20.2% and male-only studies totalled 12.7%. Only 5.3% of studies were inclusive of non-binary or transgender options for self-identification. The remaining 12.2% of studies did not include patient sex within datasets. Patient sexuality was discussed in only eight articles, in terms of psychosexual distress, sexual anxiety and esteem. Notably, a further four articles discussed sexuality only by way of age at first sexual intercourse, termed, ‘sexual debut’. However, research has not established a correlation between anomaly severity and poor sexual function or body image [21]. Extant literature in this regard has not considered the importance of other factors, such as safety and trust as relationship connectors that can support healthy sexual engagement in adulthood.

### Relationships

Despite the benefit of positive intimate connections towards self-acceptance, patient relationships were reported in less than a third (*n* = 27 or 28.8%) of the reviewed articles. Of these, 13 (13.8%) categorized relationships under: married, single, divorced, de facto, stable relationship, sexual relationship, partnered, domestic partnership, widowed, and civil union. Distinctly, patients in co-habitation with an intimate partner were found to report higher levels of psychosocial functioning irrespective of relationship category [7]. Curiously, one article reported that 35% of adults with ARM did not develop a ‘first love’ connection until after the age of 18, or not at all [22]. Therein, if patients reported not experiencing a ‘first love’ prior to age 18, the percentage of doing so after this age fell to just 9%. This delayed or absent experience of ‘first love’ may significantly impact later relationship formation, regardless of physical intimacy. While seemingly subtle, this finding underscores something fundamental: the right to love and be loved transcends medical literature’s focus on ‘sexual debut’ and society’s emphasis on physical milestones.

### Parenthood

Only seven (7.9%) studies examined parenthood, primarily focusing on delivery modes. The largest study reported on 36 patients: 21 who had vaginal deliveries and 15 who had caesarean sections. Within this cohort, two patients developed total incontinence following vaginal delivery – one after three births, and another after three births [23]. In this regard, the feasibility of vaginal deliveries in adult female patients with a history of ARM repair in childhood requires careful consideration by specialists [24]. Paternity relating to male-born adults with ARM was only discussed in three (3.1%) of the reviewed articles. These studies were focused on infertility, sperm count, motility, and quality; and patients who had attempted or become fathers. Chiefly, it appeared that the complexities of parenthood as an intersectional element beyond biological functions have been largely underreported in the literature on ARM experiences in adulthood.

### Language and spirituality

Only one study reported on the language of participants [25]. Language was intimated in a further six (6.3%) studies, exclusively for the purpose of excluding participants who could not speak English who were therefore deemed unsuitable for participation. The remaining 92.7% of studies did not report on participant languages. Spirituality of patients was not reported in (*n* = 93 or 98.9%) of the studies, making this the most significantly underreported intersectional element despite the inherent intersection between health choices and belief systems. Spirituality was briefly discussed in relation to the role of emotion-focused coping mechanisms, such as acceptance, positive-reframing, and ‘turning to religion’ [26], thereby existing as a potentially important factor for consideration in the provision of psychosocial support for adult ARM patients.

### Disability

Continence management was discussed in 33 (35.1%) of studies, predominantly intimating the use of continence systems for the management of ARM in adulthood. The most reported included colostomy, ostomy, stoma, ileostomy, and catheter. A further 12 (12.7%) of studies reported the exclusion of patients with intellectual, chromosomal, and cognitive disabilities, as determined by those studies’ eligibility criteria. Co-morbidities such as cardiac, renal, and limb malformations were reported in only 4 (4.2%) studies. This was despite associated disabilities in the adult ARM cohort reported to affect as many as 75% of patients [27]. Policy recommendations on the topic of ARM as a disability were discussed in only 4 (4.2%) studies. These discussions covered basic needs such as stoma therapy involvement in adult ARM management, counselling and financial support for medical supplies, reasonable accommodations relating to fecal incontinence in educational settings, and necessitating policy to acknowledge the condition as being a disability that affects patients across their lifespan; not just in pediatric development [21, 28–30]. Of interest, only one article discussed disability disclosure within intimate relationships [22], whereby more than half of those participants identified that telling partners about the condition was an issue they would like to address.

#### Employment and education

Education and employment were not a key focus within any reviewed studies; however, the elements were periodically captured as part of standardized survey questions. Impacts of ARM on occupational pathways, such as participants experiencing role limitations, were reportedly experienced by between 16% and 40% of the adult cohort [31]. Six (6.3%) of the reviewed studies reported on high school educational outcomes, and a further five (5.3%) studies discussed completion outcomes of university degrees; although achievement of these milestones was reportedly lower for individuals with ARM than that of the general population [32]. Five (5.3%) more studies affirmed low educational attainment as an experienced outcome related to the challenges of living with ARM in adulthood. Of interest, one study considered the transition to university, rather than completion of the milestone, to be a positive educational outcome for adult ARM individuals [33]. Only one article explored respondents’ views on outcomes that related to education and employment; finding 40% of those participants believed their condition had limited their choices in occupational and educational uptake [34]. Whether participants disclosed their condition in employment settings remained unexplored. Therefore, corresponding experiences such as role limitations, may be tied to the inherent tension between choosing to disclose in pursuit of applying reasonable accommodations and risking discrimination, versus choosing not to disclose, which in turn may limit job options.

### Mental health

Discussions regarding the mental health experiences of adults born with ARM were observable across four emergent themes as follows:

#### Mental health diagnoses

Mental health diagnoses associated with ARM in adulthood were discussed in only 8.5% of articles. Patients were described as experiencing lifelong psychological complications such as depression [35], with stoma presence reported to pose a serious psychological handicap [36]. Female participants experiencing a history of surgical intervention were reported to have higher rates of anxiety and depression [37]. One article reported that psychological ill-effects relating to the physical condition occurred in 45.1% of patients overall [38]. However, another article reported that only 38% of patients’ psychological needs were effectively identified [28]. Of interest, participants with certain mental health diagnoses such as schizophrenia, autism spectrum disorder, and severe personality disorder were commonly excluded on the premise of those study’s eligibility requirements. Beyond this rationale, no further indication was reported as to the necessity of employing exclusionary criteria on this basis [39].

#### Emotional wellbeing

Emotional wellbeing was discussed in only 8.5% of the reviewed studies. Conversely, 73% of participants in one article reported that bowel issues had a negative effect on their emotional wellbeing [40]. Additional contributors to negative effects included continued, medicalized attention to the genital region by way of anal dilations, wash outs, and urinary tract infections [38, 41]. Further data discussed depressive feelings experienced by adult male patients with ARM, due specifically to difficulties within intimate sexual relationships [22]. This distinct gap between high patient reporting of negative effects on their emotional wellbeing and perceivably low uptake of literature prioritizing this occurrence, may offer insight into maladaptive coping strategies observed in the absence of therapeutic intervention.

#### Maladaptive coping strategies

Maladaptive coping strategies were present within 6.3% of the reviewed articles and comprised a diverse range of adaptations made by adult ARM patients living with the condition. Substance misuse was termed to include misuse of alcohol, tobacco, and soft drugs [22]. The highest reported rate of substance misuse was 31% however; it was not considered an ‘important trigger’ by surgeons [38]. Feelings associated with maladaptive coping included depression, embarrassment, and negative self-perception [42]. In the absence of psychosocial support, feelings appeared to intensify through denial, self-blame, and behavioural disengagement [27].

#### Mental health interventions

**Fig. 4:**
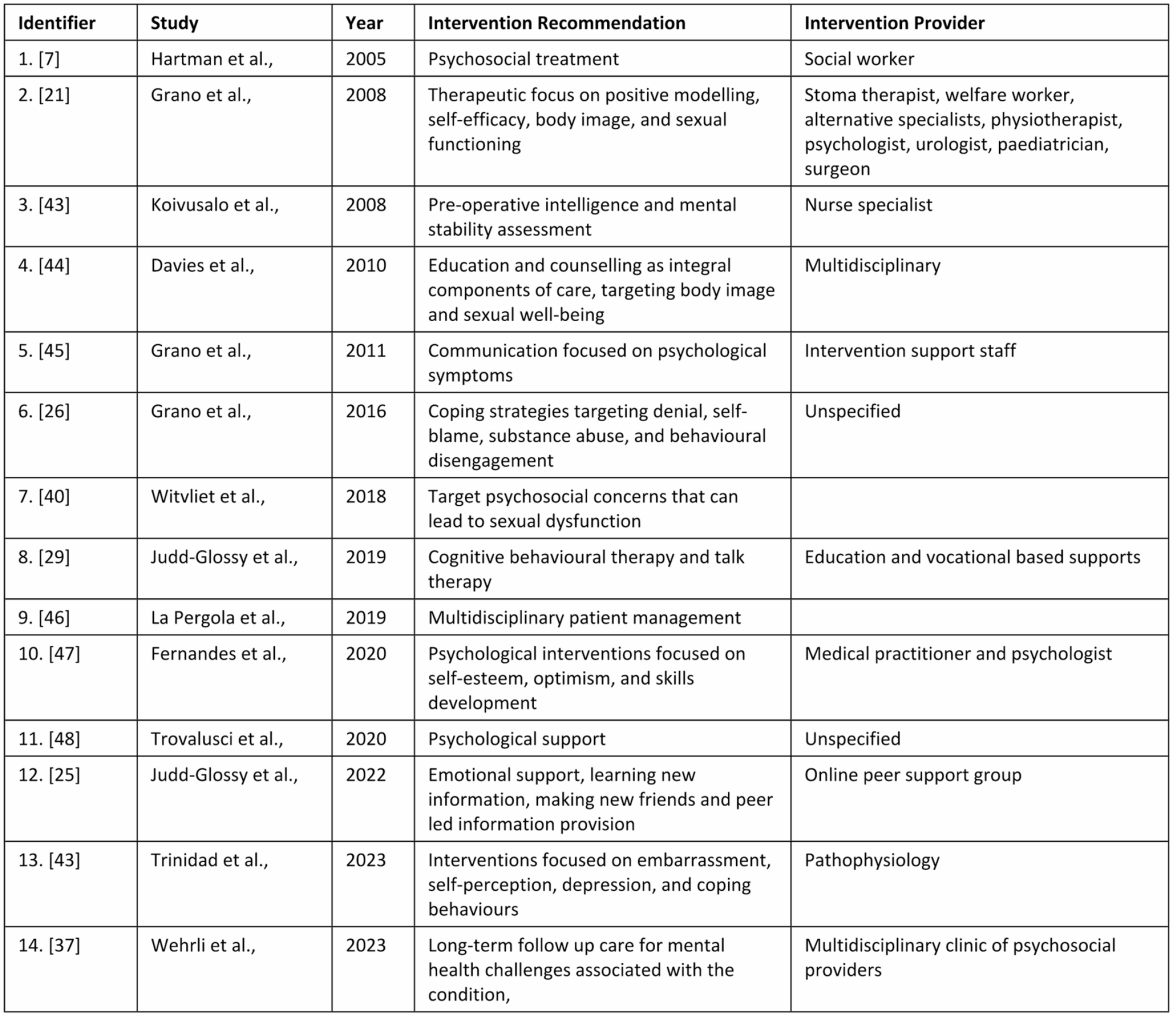
Mental health intervention recommendations from the reviewed studies Multidisciplinary. including psychologist and social worker Multidisciplinary, including psychologist

Although nearly half (46 or 48.9%) of the reviewed studies revealed mental health comorbidities such as anxiety and depression as factors concerning adults living with ARM, interventions for such concerns were mentioned in only (14 or 14.8%) of the literature base. Furthermore, the majority of those were recommendations only, rather than applied therapeutic interventions. Recommendations included skills-based psychological interventions that focused on self-esteem, multidisciplinary approaches to health service delivery, and to provide counselling and education as an integral part of ongoing ARM mental health management indicated in Fig. 4.

Notably, such recommendations were not considered in the literature base until the year 2005, despite literature being identified as far back as the year 1986. In addition, little activation of mental health support, intervention, or referrals has occurred for adult ARM patients to date. This potentially contributed to the cohort of adult ARM individuals identifying their need for mental health support was not being fully met by health care professionals, thereby creating their own online peer support group; as identified in a 2022 study [25]. In this manner, activation has begun to take place in the form of information provision, health literacy, and peer-to-peer support for previously isolated individuals. It is of particular interest to the current study and critical social work lens, that two such studies within the literature base recommended social work practice as a specialist contributor to the multidisciplinary approach; required to effectively support adults living with this condition [7, 40]. A remaining (5 or 5.3%) studies acknowledged the presence of mental health co-morbidities within the adult population living with ARM; however, no mitigating interventions or recommendations were discussed therein.

A lack of psychologically informed research designs was also identified. Only one of the 94 articles included for review was psychologically focused; examining adult psychosexual wellbeing after pediatric surgery for ARM [41]. Interestingly, 50% of participants from the psychologically focused study felt that experiences of sexual dysfunction were not related to their anatomical malformation or severity, but rather, experiences of dysfunction were psychologically embedded, and therefore required psychological amelioration alongside medical care. Of particular importance, the study was informed via patient’s self-reporting of psychosexual experiences; rather than the bulk of the literature base that employed an analysis of patient medical records and expected outcomes. The rationale given for this predominant approach of reviewing patient medical records, rather than obtaining patient experiences in a qualitative manner, was reported to be the difficulty in locating or gaining participants from the adult cohort. This contrast between only one psychologically focused article, yet al.most half (46 or 48.9%) of the literature indicating the presence of mental health co-morbidities, demonstrated an acknowledgement of mental health concerns being experienced by the adult population, but also a lack of research dedicated to informing interventions for mental health support.

More than half of the reviewed studies (51 or 54.2%) utilized a medical lens and were predominantly concerned with anticipated continence outcomes for adult ARM patients following pediatric surgery for the condition. Psychosocial theories were present in only six (6.3%) studies yet introduced a range of considerations to support adult patients, such as transactional theory, emotional coping, and positive psychology. The remainder of studies included for review did not discuss any mental health interventions, recommendations, or support strategies for adult patients living with ARM; despite the numerous reported symptoms associated with the condition including:

1. Substance misuse [22, 38].
2. Lifelong psychological concerns including depression [35].
3. Depression and anxiety associated with surgical intervention [37].
4. Negative impacts on well-being [46, 50].
5. Stoma presence posing serious psychological concern [49].
6. Negative impacts on psychosexual health and body-image [51].
7. Embarrassment and depression [52].
8. Depressive feelings surrounding sexual and close relationships [53].
9. Feelings of shame [54].

Mental health co-morbidities experienced by adult ARM patients were therefore acknowledged in the reviewed literature however, a lack of further investigation into how patients are coping or not coping with these symptoms was also observed. Knowledge and research gaps in this field present an appropriate space for the role of social work in conducting future studies, amplifying patient voices, and contributing to multidisciplinary interventions aimed at improving psychosocial functioning impacted by the condition.

### Dataset 2.0: psychosocial challenges and supports

Psychological and sociological experiences related to living with ARM in adulthood were thematically coded and reported in order of theme prevalence. In the interest of shifting away from deficit and diagnosis-focused paradigms, both psychosocial challenges and supports were identified from within the literature base.

### Psychological challenges

Reported psychosocial challenges experienced by adults born with ARM were observable across six emergent themes in the literature base as follows:

#### Psychosexual challenges

Psychosexual challenges were present in (*n* = 18) studies and covered a range of patient concerns. These included that adult individuals with ARM achieved fewer psychosexual milestones and held significantly lower psychosexual development scores than general population samples [22]. Patients with frequent incontinence experienced heightened sexual anxiety, and sexual intercourse was reported at times to worsen the physical and psychological impacts of the condition [44].

#### Lack of mental health support

A lack of mental health support was indicated in (*n* = 13) studies. Discussions included the need for more paramedical and psychological care [22]. One article cited the most common psychological impacts as depression 87%, anxiety 85%, and post-traumatic stress disorder 46% [29], alongside limited knowledge by professionals around the psychological impacts of ARM in adulthood [55]. In addition, another article suggested that one third of all adult ARM participants needed psychological support within the last two years [28].

#### Patient disengagement

Patient disengagement with health care services for their condition was observable within (*n* = 11) studies and appeared in relation to refusal of subsequent surgical interventions [56–58], absconding from follow up care [59], and a lack of coping with the condition, including negative self-perception [60].

#### Impaired psychological functioning

Impacts on psychological functioning were discussed in (*n* = 8) studies and appeared via a range of phenomena. These included adults with ARM not experiencing ‘normal’ bowel behaviours, rendering an unawareness of the condition as it related to serious psychological handicaps when managing external systems such as stomas [36]. Severe anxiety was detected in relation to pelvic examinations [61], and data suggested psychological functioning was impacted negatively by self-perceptions of condition severity, irrespective of accuracy [45].

#### Impacts on emotional wellbeing

Negative impacts on emotional wellbeing were mentioned in (*n* = 7) of the reviewed studies. Impacts included impaired body image, feelings of depression and shame [27, 31]. Alongside lowered levels of emotional functioning of the adult cohort when compared to the pediatric cohort [62], and lowered levels of emotional wellbeing and overall energy [38].

#### Inhibitors on self-efficacy

Inhibitors on self-efficacy were present within (*n* = 5) of the reviewed studies. Adult ARM patients who were discouraged from taking active roles in their health journey during adolescence, often due to ascendant parental involvement, were found to have inhibited development of self-efficacy essential for navigating the condition in adulthood [63]. The age of pediatric surgeons attending to adult patients with ARM was also reported to influence outcomes in self-efficacy. Older surgeons (< 55) were less likely to consult with adult ARM patients than their (> 44) colleagues, despite the lack of providers knowledgeable in the condition available in adult health sectors [64]. Poorly managed post-operative care was also found to correlate with poorer self-efficacy outcomes during times when patients were feeling their most vulnerable [21]. The remaining (*n* = 32) articles included for review did not discuss psychological challenges experienced by adults living with ARM; despite the significantly high occurrence of mental health comorbidities such as anxiety and depression associated with the condition.

### Psychological supports

Reported psychological supports experienced by adults born with ARM were observable across four emergent themes in the literature base as follows:

#### Psychological involvement

Psychological involvement was identified in (*n* = 14) of the reviewed studies. Involvement specifically included efforts that focused on psychological treatment for body image and emotional distress [62], and a multidisciplinary approach to patient evaluation, inclusive of psychology [46]. Cognitive behavioural therapy and talk therapy were the most applied therapeutic modalities used with adult ARM patients, with one article citing usage percentiles of 51% and 82% respectively [29].

#### Independence stimulators

Factors that stimulated independence were discussed in (*n* = 7) studies. Coping strategies that reduced self-blame, substance misuse, behavioural disengagement and denial were found to enhance individual’s health related quality of life [26]. When patients became more independent at a younger age, they were found to demonstrate a greater sense of discipline and prioritise independence later in life [32]. Participation in online peer support groups was also found to improve patient access to health information and improve overall mental health experiences associated with the condition such as anxiety, depression and isolation [25].

#### Health literacy

Provider and patient health literacy was an identified support within (*n* = 7) studies. Provider prioritization and preservation of sexual function for sexual activity was closely aligned with improved psychosocial functioning and development [52]. Considerations included developmentally appropriate timing of discussions with patients around reproductive health [30]. One article found that the development of practitioners’ health literacy surrounding the condition was of importance to 96% of patients, including the implementation of ‘soft skills’; due to the highly sensitive nature of ARM [28].

#### Positive empowerment

Positive empowerment was an identified support in (*n* = 6) of the reviewed studies. Findings indicated social supports such as companionship, esteem-support, and support in everyday problem-solving enhanced individuals’ overall quality of life and psychosocial functioning [65]. Adult ARM patients who felt more empowered in relation to their bodies felt less embarrassment and shame about the condition [21]. In addition, patients who felt psychologically empowered, and sought earlier follow-up care in adulthood reported positive impacts in their lives, such as the desire to spend more quality time with their families and children [54]. The remaining (*n* = 60) articles did not discuss any psychological supports that assisted adults navigating life with ARM, demonstrating the significant research gap in prioritizing and understanding this aspect of their lived experiences.

### Sociological challenges

Reported sociological challenges experienced by adults born with ARM were observable across six emergent themes in the literature base as follows:

#### Navigating adult health care systems

Difficulties in navigating adult health care systems were indicated in (*n* = 19) of the reviewed studies. Discussions included changed surgical procedures over time, budgeting restraints, and treatment expenses reflecting changes in quality-of-life outcomes experienced by the cohort [36, 62]. In addition, cost effectiveness was queried in relation to the effectiveness of interventions [66].

#### Experiences of incontinence

Bowel or urinary incontinence experienced in social settings was discussed in (*n* = 15) studies. Continence rates amongst the adult ARM population were reported to be unsatisfactory, which caused a significant number of social problems. One article indicated that 85% of participants felt the condition was indeed a social disability [67]. Considerable time, energy, and effort were required by participants for the daily management of the condition [68]. However, despite patient dedication, continence rates were ‘not optimal’, with one article reporting 34.8% of adult patients experienced fecal incontinence, and 71.4% experienced urinary incontinence; negatively impacting their social participation [31].

#### Negative experiences within family systems

Negative experiences within family systems were indicated in (*n* = 12) of the reviewed studies. This occurred by way of patient exposure to family negligence of their condition, including low levels of health literacy and education being transferred from providers to patients [69]. Tensions between parents of patients with ARM during childhood reportedly contributed to delayed sexual development during adolescence and adulthood. Furthermore, anal dilations, wash outs, and recurrent urinary tract infections could become associated with pain, disrupting the genital region as a sexual organ; more commonly associated with arousal in the wider adult population [41].

#### Interpersonal challenges

Challenges within interpersonal and intimate relationships were described in (*n* = 11) studies. Findings indicated that feelings of demoralization contributed to adults with ARM avoiding or reducing their participation in relationship building; which contributed in some cases to depressive episodes [70]. Instances of marriage lacking sexual intercourse were also reported, with sexual function and intimacy remaining poorly investigated. Studies indicated adult patients are in need of proper psychosocial assessment, information provision, and follow-up management to address these difficulties [39, 71].

#### Professional, and social resourcing restraints

Restraints on professional and social resourcing were discovered in (*n* = 10) studies. Examples included patients predominantly working in sedentary occupations due to their condition, which led to further sub-optimal outcomes in social integration and networking [32]. Patients also expressed their desire for increased social and health related support groups, to help with social empowerment and questions relating to condition symptoms and management [30].

#### Resilience inhibitors

Resilience inhibitors were detected in (*n* = 8) studies. Findings suggested that patients whose childhood continence concerns were ignored or denied, were found to have increased social problems later in life such as bowel enema dependence, repeated soiling, involuntary flatulence, and fixation on constant toilet access in social settings [72]. Overprotection by parents of children with ARM was also found to inhibit the personal resilience required to navigate the condition as an adult, with disease cognition becoming distorted. This in-turn influenced negative changes to self-perception and socialization for adult patients [22]. The remaining (*n* = 19) studies did not report on sociological challenges experienced by the adult ARM population, despite the condition being inherently tied to all aspects of an individual’s lived experience in social settings, participation, and life choices.

### Sociological supports

Reported sociological supports experienced by adults born with ARM were observable across four emergent themes in the literature base as follows:

#### Establishing positive relationships

Establishing interpersonal and intimate relationships positively influenced social support construction in (*n* = 16) of the reviewed studies. Sexual health and sexuality arising from these influences attributed to social, mental, and emotional wellbeing, even with the presence of physical dysfunction [27, 48]. Inclusive family dynamics and family life were also mediating factors associated with higher levels of psychosocial functioning for individuals navigating ARM in adulthood [55, 73].

#### Participation and networking

Patients and health care providers who participated actively in networking were an identified support indicated in (*n* = 14) studies. Patient-specific factors included seeking emotional support from contacts, and in rare cases, turning to spiritually based social systems such as religion [62]. Profession-specific factors included pediatric surgeons that participated in registers aimed at supporting ARM patients more comprehensively [74]. Health professionals that networked actively were more involved with other departments, such as urology and gynaecology; thereby improving evaluation and management for adults with ARM [69].

#### Optimism for the future

Optimism for the future was identified as a supportive social construct in (*n* = 14) studies. Examples included optimistic views of one’s life, of oneself, and the future; partially mediating the effects of incontinence on the individual’s overall quality of life [47]. Experiences relating to improved social outcomes in adulthood following surgical revision for ARM were described by patients as being able to stand for longer periods of time at work and during social commitments, and with the integration of medical intervention, alongside social supports, a balance of optimisation was achievable between these two mediators [32, 54].

#### Achievement of developmental milestones

The achievement of developmental milestones was an emergent theme in (*n* = 13) studies. Parental support in health care settings was appreciated by adolescent individuals with ARM, and specifically; when discussions centred towards pathways to follow-up care in adulthood [63, 72]. Positive social and relational experiences were reported to improved patients’ communication skills regarding the condition, and their ability to cope with the condition socially [45]. The remaining (*n* = 37) studies did not report on sociological supports that may mitigate negative effects associated with the lived experience of ARM in adulthood.

## Discussion

Being born with anorectal malformation is a rare human phenomenon. The experiences and outcomes related to living with the condition in adulthood remain significantly unexplored. Therefore, this systematic quantitative literature review aimed to explore and report on patient intersectionality, in terms of what elements were prioritized or not prioritized within the literature base. Mental health comorbidities and intervention recommendations were also identified, alongside expected or experienced psychosocial challenges and supports faced by individuals navigating this condition in adulthood. The lived experiences of ARM including stressors of anxiety and depression which can accompany the condition, were indicated in the literature base. However, the adult cohort remained significantly underrepresented, or alternatively, represented within pediatric journals that were primarily authored by paediatric surgeons; and focused predominantly on continence outcomes. Such outcomes neglected the complex psychological and sociological nature of the condition and were often forecast at the time of pediatric presentation. Notably, adult patients sought clinical review at an average age of 30.2 years, far exceeding the Krickenbeck (2005) recommendation of a 10-year maximum follow up. This demonstrates the need for longitudinal studies to identify adult-specific outcomes.

Intersectional elements such as cultural and class systems have rarely been considered in previous literature as to their effects on health literacy or follow-up care. Yet, societally conditioned expectations such as marriage influenced patients’ timing to health care re-access for the condition in adulthood; particularly for women [52]. Importantly, increased anatomical severity of the condition did not necessarily correlate with reduced sexual function for any sex, with positive intimate encounters supported more strongly through relational trust, and healthy body image. Following the development of intimate partnerships, parenthood was rarely reported on or was reported via discussions of adults with ARM as children of parents, rather than being envisaged as parents themselves. These findings reaffirm the importance of upholding the individual’s right to self-determination, bodily autonomy, and opportunities to achieve or simply access developmental milestones. This may assist in mitigating the effects of uncomfortable conversations with parents, hospital visits, and the condition itself. How the condition was navigated alongside parents, doctors, and eventually intimate partners, was also an observed phenomenon that required trust and understanding. However, whether an individual made the decision to disclose their diagnosis in relationships, or employment settings, remained significantly unexplored. This is despite disclosure potentially leading to accommodations, and therefore, needs being met. Versus non-disclosure, whereby accommodations may not be made if not known; thereby limiting intimate connectedness and job options or preferences. Both of which are significant experiences in the lives of all humans.

An observable gap existed between patients reporting negative impacts on their mental and emotional wellbeing, and a perceivably low uptake of literature prioritizing this experience. Data extracted from the literature base indicated that in the absence of therapeutic interventions; feelings of distress, depression, and anxiety were observed to intensify for individuals. Patients appeared to be distinctly aware of the importance of psychological support for this condition, believing areas such as sexual dysfunction to be more strongly related to thoughts and feelings about the body, rather than the anatomical malformation of the body. However, only one article was psychologically focused, juxtaposed with almost half of the reviewed literature describing some form of mental health concern or diagnosis being experienced by the adult cohort. This research gap may be explained by most of the extant literature using a biomedical lens concerned with surgical intervention and associated continence outcomes but consequently neglecting psychosocial implications.

Despite this overwhelming occurrence of reported mental health challenges such as anxiety and depression coinciding alongside the lived experience of ARM in adulthood, over one third of reviewed articles did not discuss psychological concerns, and more than half did not discuss any recommendations for psychological support or intervention. Making this the largest area of invisibility from within the literature base and demonstrating the significant research gap in prioritizing this experience. This systematic quantitative literature review has therefore provided an awareness of the intersectional and psychosocial complexities of ARM in adulthood that future research can investigate further and expand upon. In addition, the systematic review process highlighted the benefits of adopting an intersectional approach to understanding lived experiences of navigating lifelong health conditions as a justified and versatile research design.

### Potential research limitations

Although the systematic quantitative literature review methodology holds many advantages over other review styles such as narrative approaches, limitations can still exist. Studies included in this systematic review were required to be available in full-text English, which meant articles that may have met set eligibility requirements, but were not available in full-text English, were not included for review. Every effort was made to provide visibility of such studies, with non-English articles included in PRISMA screening, and not excluded prior to this important recording of results.

The systematic review process is also intended to be reproducible. However, categorising studies into a framework of quantitative datasets required the author to employ a level of interpretation and decisiveness as to what categories data most appropriately fell into, which can impact the conclusions made about discovered themes. For example, psychological, and sociological domains were categorised separately in order to make data input manageable. However, domains also commonly overlap, whereby decisions were required in allocating elements and experiences to the corresponding domain category. Notwithstanding these potential research limitations, the systematic quantitative literature review provided a transparent and robust review process, towards generating further interest in this field.

## Conclusion

This systematic quantitative literature review demonstrated an overwhelming prioritization of studies focused on forecasting long-term continence outcomes for adults, that were primarily based on type and severity of ARM at the time of paediatric presentation. A distinct lack of longitudinal research or adult patient interviews, combined with an idle recommendation of follow up not exceeding 10 years post-operatively; has rendered a population of ARM survivors invisible and their outcomes in adulthood largely unknown. Biological circumstances of the condition have been indicated by the literature base alongside improvements in surgical techniques that have occurred over time. However, this focus, teamed with the predominance of medically siloed viewpoints, has thus far neglected patient intersectionality and psychosocial experiences of living with the condition. For this reason, the current study maintained its’ focus on investigating demographic representation or non-representation, mental health co-morbidities, and psychosocial experiences of living with ARM in adulthood.

Findings indicated an overwhelming underrepresentation of patient intersectionality, mental health co-morbidities and therapeutic interventions, psychosocial experiences and outcomes. According to reporting embedded within the literature base, both psychological and sociological challenges outweigh the number of identifiable supports. Mental health challenges associated with experiencing the condition remained the most distant from supportive factors, requiring psychosocial interventions in future health care service and delivery. Even in cases of severe anatomical presentation, adult patients believed their challenges aligned more strongly with mental health and psychosocial functioning, and with a lack of support and understanding from health care providers. Very little research currently investigates the importance of these concepts, despite the reported psychological impacts of the condition and lack of transitional care from pediatric to adult health services.

Ongoing research should prioritize the lived experiences of adult individuals born with ARM, as this cohort offers invaluable and nuanced perspectives in defining what it means to navigate the condition, and the limited adult health services specialising in this field. The promotion of identified supports can therefore be enacted by allied health professionals, such as social workers and psychologists. In this manner, multidisciplinary approaches can support individuals with therapeutic modalities that prioritize patient-led care, improved psychosocial functioning, and participation in life.

## Data Availability

The datasets generated that support the findings of this study are not publicly available. The data are, however, available from the authors upon reasonable request and with the permission of Griffith University Human Research Ethics Committee, School of Allied Health, Sport, and Social Work.
